# Enhancing Brassinosteroid Signaling via Overexpression of Tomato (*Solanum lycopersicum*) *SlBRI1* Improves Major Agronomic Traits

**DOI:** 10.3389/fpls.2017.01386

**Published:** 2017-08-10

**Authors:** Shuming Nie, Shuhua Huang, Shufen Wang, Dandan Cheng, Jianwei Liu, Siqi Lv, Qi Li, Xiaofeng Wang

**Affiliations:** ^1^State Key Laboratory of Crop Stress Biology for Arid Areas, College of Horticulture, Northwest A&F University Yangling, China; ^2^Qinghai Key Laboratory of Vegetable Genetics and Physiology Xining, China

**Keywords:** agronomic traits, Brassinosteroids, nutritional quality, signaling regulation, *SlBRI1*, tomato

## Abstract

Brassinosteroids (BRs) play important roles in plant growth, development, and stress responses through the receptor, Brassinosteroid-insensitive 1 (BRI1), which perceives BRs and initiates BR signaling. There is considerable potential agricultural value in regulating BR signaling in crops. In this study, we investigated the effects of overexpressing the tomato (*Solanum lycopersicum*) *BRI1* gene, *SlBRI1*, on major agronomic traits, such as seed germination, vegetative growth, fruit ethylene production, carotenoid accumulation, yield, and quality attributes. *SlBRI1* overexpression enhanced the endogenous BR signaling intensity thereby increasing the seed germination rate, lateral root number, hypocotyl length, CO_2_ assimilation, plant height, and flower size. The transgenic plants also showed an increase in fruit yield and fruit number per plant, although the mean weight of individual fruit was reduced, compared with wild type. *SlBRI1* overexpression also promoted fruit ripening and ethylene production, and caused an increase in levels of carotenoids, ascorbic acid, soluble solids, and soluble sugars during fruit ripening. An increased BR signaling intensity mediated by *SlBRI1* overexpression was therefore positively correlated with carotenoid accumulation and fruit nutritional quality. Our results indicate that enhancing BR signaling by overexpression of *SlBRI1* in tomato has the potential to improve multiple major agronomic traits.

## Introduction

Brassinosteroids (BRs) are a group of steroid phytohormones that play important roles in plant growth, development, and stress responses (Zhu et al., 2013; Xia et al., 2015). Studies to date, mostly using the model plant *Arabidopsis thaliana*, have shown that BRs bind to the receptor Brassinosteroid Insensitive 1 (BRI1), activating it and initiating a signal transduction cascade. BR-deficient or BR-insensitive mutants generally have serious developmental deficiencies, such as dwarfed stature, dark green leaves, short petioles and hypocotyls, delayed flowering and senescence, reduced male fertility, decreased seed setting rates and photosynthetic capacity, as well as perturbed phytohormone balances. Furthermore, BR-insensitive mutants have been reported to have increased transcript levels of BR biosynthetic genes, as well as a higher BR content (Chory et al., 1991; Li and Chory, 1997; Noguchi et al., 1999; Wang et al., 2008; Kim and Wang, 2010).

Brassinosteroids influence many important agronomic traits associated with growth, photosynthesis, architecture, and yield and crop quality. The application of BR analogs has been shown to promote plant growth, photosynthesis, fruit carotenoid accumulation, and quality attributes of fruit, while inhibiting BR biosynthesis with the compound Brassinazole has the opposite effect (Yu et al., 2004; Wu et al., 2008; Xia et al., 2009a). BRs increase photosynthetic capacity by activating RuBisCO activase (Xia et al., 2009a), and BR treatment induces ethylene production in fruit by inducing the expression of ethylene biosynthetic genes, thereby regulating ripening (Zhu et al., 2015). In addition, exogenous BRs have been shown to enhance resistance of plants to abiotic and biotic stresses, such as drought, low and high temperatures, high salinity, and pathogen or nematode attack (Collins, 2007; Xia et al., 2009b; Ahammed et al., 2012; Xi et al., 2013).

However, in an agronomic context, BR application is very expensive and its effects are unstable, which have limited its potential value in crop production. An alternative strategy is the genetic manipulation of BR biosynthesis or signaling is to alter BR content or its signaling intensity in crop plants (Wang et al., 2016). Overexpression of *DWARF*, a BR biosynthetic gene, in *A. thaliana*, tomato (*Solanum lycopersicum*), rice (*Oryza sativa*), and maize (*Zea mays*), has been reported to result in higher endogenous BR levels, increased growth, nutrition quality and yield (Wang et al., 2014; Li et al., 2015; Eremina et al., 2016). Furthermore, overexpression in tomato of *AtBZR1-1D*, a transcription factor from *A. thaliana* involved in BR mediated signaling, increased carotenoid accumulation and other quality attributes in fruit (Liu et al., 2014), while constitutive activation of BR signaling enhanced freezing resistance by regulating cold responsive gene expression in *A. thaliana* (Eremina et al., 2016). Accordingly, there is considerable interest in enhancing endogenous BR levels or associated signaling as a means to improve crop yield, nutritional quality, and stress tolerance (Divi and Krishna, 2009).

The perception of BRs by BRI1 and consequent BR signal transduction in *A. thaliana* has been investigated using biochemical, genetic, and proteomic approaches (Ryu et al., 2010). Endogenous overexpression of *A. thaliana AtBRI1* was reported to cause in an increase in petiole length and sensitivity to BRs (Fujimura and Samet, 2001), while overexpression of *TaBRI1* (*Triticum aestivum*) in *A. thaliana* resulted in early flowering, greater silique size and seed yield, and an increase in root sensitivity to BR (Singh et al., 2016). The phenotype of the tomato *bri1* (*cu-3*) mutant is similar to that of the *A. thaliana bri1* mutant, and constitutive overexpression of *SlBRI1* in tomato was reported to rescue the *cu-3* dwarf phenotype (Koka et al., 2000; Holton et al., 2007). Furthermore, the transgenic lines had longer internodes and hypocotyls, and their leaves were more ovoid and showed reduced serration compared to the wild type (WT) (Koka et al., 2000; Holton et al., 2007). However, when *SlBRI1* was overexpressed in the *A. thaliana AtBRI1* mutant (*bri1-5*), it did not fully complement the mutation (Holton et al., 2007), suggesting that the functions of different *BRI1* orthologs may vary somewhat; an idea that needs to be further investigated.

To date, the potential for using *SlBRI1* and BR signaling in tomato to enhance major traits related to architecture, yield, and fruit nutritional quality has not been determined. In this current study, we generated *BRI1*-overexpressing tomato plants exhibiting increased BR signaling intensity and evaluated seed germination, growth, developmental characteristics, fruit nutritional quality and yield. Based on these results, we discuss the feasibility of improving tomato growth and fruit nutritional quality by genetic manipulation of *SlBRI1*.

## Materials and Methods

### Plant Material and Growth Conditions

For the seed germination experiment, seeds from *S. lycopersicum* (cv. Micro-Tom; used here as the WT), and from the T2 generation transgenic lines (see below for details of generating the transgenic plants), were imbibed on Petri dishes with 0.74% agar and maintained at 28°C for 3 days in darkness, and then maintained at 25°C for 4 days under a photosynthetic photon flux density (PPFD) of 200 μmol m^-2^ s^-1^. For the phenotype evaluation, seeds were germinated at 28°C in Petri dishes lined with two layers of filter paper moistened with deionized water. The germinated seeds were then sown and plants were also grown in plastic pots (7 cm × 7 cm × 8 cm), filled with 105 g of a mixture of peat and vermiculite (v/v = 7:3) in trays. Seedlings and plants were grown in growth chambers, at a temperature 25/20°C, a PPFD of 500 μmol m^-2^ s^-1^, and a 12-h photoperiod. Flowers were tagged at the full-bloom stage to synchronize developmental comparisons. Fruits were identified as being at the breaker (B) stage when they displayed the first sign of external color change. Fruit from the WT and transgenic lines took 54 and 45 days, respectively, to develop from anthesis to the B stage. Fruit were considered to be at the mature green (MG) stage 3 days before B, while fruit at 3 days after B were considered to be at the P (pink) stage and those at 6 days after B were at the MR (mature red) stage.

### Constructs and Plant Transformation

The tomato *SlBRI1* gene was PCR-amplified from tomato (*S. lycopersicum* cv. Micro-Tom) cDNA based on a UniGene sequence (Solyc04g051510), and a Flag peptide encoding sequence was added to the 3′ end. The *SlBRI1* cDNA sequence was cloned into the *Kpn*I and *Xba*I sites of the binary pBI121vector, which carries the kanamycin resistance gene for bacterial and plant selection, and drives transgene expression using the constitutive cauliflower mosaic virus 35S promoter. The construct was transformed into the *Agrobacterium tumefaciens* strain GV3101 by electroporation, which was then used to transformed tomato cotyledon explants (Park et al., 2003). Three homozygous transgenic tomato lines (*BRI1*: OX4, *BRI1*: OX6, and *BRI1*: OX37), exhibiting high *SlBRI1* expression levels, were chosen for subsequent phenotypic and molecular characterization.

### CO_2_ Assimilation Rate Detection

The CO_2_ assimilation rate (Pn) was determined using a portable photosynthesis system (LI-6400-40, Li-Cor, Lincoln, NE, United States). All measurements were carried out at 400 μmol (CO_2_) mol^-1^, at 22°C, and 800 μmol m^-2^ s^-1^ light intensity.

### Phenotypic Characterization

Plant height, expansion diameter of plant, leaf area, length and width of the third leaf were determined at 30 days after sowing and of the sixth leaf at 60 days after sowing. The fresh weight (FW) of the shoot, leaf mass per area, leaf thickness, stigma length, and petal length were measured at 30 days after sowing. The third and sixth leaves were photographed with a digital camera (Canon G15, Japan) at 30 and 60 days after sowing. Leaf areas were calculated using ImageJ (Collins, 2007) software. Leaf thicknesses of WT and transgenic plants were measured using light microscopic observation of paraffin sections, generated as in Yang et al. (2011), with an Olympus microscope (BX53, Japan). The third leaf was selected and at least six sections were analyzed. The growth and fruit characteristics of 15 plants per transgenic line and of the WT genotype were observed.

### Ethylene Detection

To measure ethylene production, B, P, and R stage fruit of approximately the same size were harvested and kept in sealed plastic bags for 3 h at room temperature (each biological replicate corresponded to five fruits). One milliliter of gas was removed from each plastic bag with a syringe, and ethylene production was determined using a gas chromatograph, which main specifications were FID detector, DB-130 m, 0.32 μm, 0.25 μm chromatographic column (Agilent 7890B, United States). All samples involved three biological replicates.

### Gene Expression Analysis

RNA from various organs of WT and transgenic plants was extracted using the RNAiso Plus (TaKaRa, Japan) kit, according to the manufacturer’s instructions. Residual DNA was removed with DnaseI (Thermo, United States). One microgram total RNA was reverse-transcribed using a cDNA synthesis Kit (Roche, Switzerland), following the manufacturer’s instructions. The cDNA concentrations were normalized to the expression levels of the tomato ubiquitin gene (X58253.1) for subsequent RT-PCR analysis. The qPCR experiments were performed using a Real-Time PCR system (Bio-Rad) with SYBR Green Master Mix (Vazyme, Nanjing, China). Relative gene expressions were calculated using the 2^-ΔΔCT^ method (Livak and Schmittgen, 2001) and the tomato *UBI3* gene was used as the internal control. The experiment had three independent biological samples per line and each sample had four technical replications. Gene-specific qPCR primers are listed in Supplementary Table S2. The WT and transgenic plants were represented by four replicates for each sample.

### Determination of Carotenoid Content

Carotenoids were extracted and analyzed as previously described (Liu et al., 2014) with modifications. Tomato pericarp (0.5 g) was ground to a fine powder in liquid nitrogen and extracted with 15 mL of hexane/acetone/ethanol (1:1:1) solution containing 0.01% butylated hydroxytoluene (BHT) on a magnetic stirrer for 30 min. The extracts were centrifuged at 4000 × *g* at 4°C for 10 min, and the supernatant transferred to 50 mL tubes. Fifteen milliliter of hexane/acetone/ethanol was added to the sediment and the extraction process was repeated. The supernatants from the two extractions were merged, before hexane was added up to a total volume of 25 mL. The absorbance of 5 mL of the mixture was measured at 450 nm (UV-1750; Shimadzu Corporation) and this value used to represent total carotenoid levels. The remaining mixture was frozen and evaporated using a vacuum evaporator (RE-52AA). The residue was dissolved in 1 mL ethyl acetate and 20 μL were injected onto a high pressure liquid chromatograph (HPLC, Shimadzu [LC 2010A, Japan]) using an autosampler. All the procedures were performed under dim light. A Nova-Pak C18 column (4.6 mm × 150 mm) and ultraviolet detector were used with a mobile phase of methanol/acetonitrile (45/55 by volume) at a flow rate of 1 mL/min. The total retention time was 25 min. Absorbance was detected at 450 nm. Standards (lutein, lycopene, and β-carotene; Sigma, United States) were prepared and used to identify and quantify the corresponding carotenoids. The carotenoid content was expressed as μg/g FW of tomato fruit.

### Western Blot Analysis

Proteins was extracted from young leaves (0.2 g) of 25-day-old transgenic and WT plants with 2 × SDS gel loading buffer, as in Wang et al. (2016).

### Fruit Firmness

To measure fruit firmness, R stage fruits of the approximately same size and location were harvested (each biological replicate consisted of five fruits) and firmness was determined with a hand-held penetrometer (FHM-1, Japan). Data values are the means ± SD of six independent biological samples.

### Soluble Solid Contents

Soluble solid contents (^o^BRIX) of total fruit juice from freshly harvested R stage fruits were determined with a hand-held refractometer (Chengdu Optical Instrument Factory, Chengdu, China). Data values are the means ± SD of six independent biological samples.

### Soluble Sugar Contents

The soluble sugar contents were measured as previously described (Liu et al., 2014). The experiment had three independent biological samples per line and each sample had four technical replications.

### Ascorbic Acid Contents

Ascorbic acid contents were determined as previously described with minor modifications (Hu et al., 2016). Frozen pericarp (1 g) of R stage fruits was ground to a fine powder in liquid nitrogen and extracted with 5 mL of 6% (w/v) trichloroacetic acid (TCA). The homogenates were centrifuged at 4000 × *g* for 15 min at 4°C. The supernatant (0.4 mL) was transferred to a 10 mL tube containing 0.4 mL of 5 mM DL-dithiothreitol (DTT) in 0.4 M potassium phosphate buffer, pH 7.4. The 10 mL tube was incubated for 30 min at 37°C, and 0.2 ml 0.5% (w/v) *N*-ethylmaleimide (NEM) added, followed by incubation for 1 min at room temperature. Color reagent (1.6 mL) was then added to the mixture, which was incubated for 1 h at 37°C. The absorbance was recorded at 550 nm (Shimadzu UV-1750). The color reagent was prepared as follows: solution A, 31% orthophosphoric acid, 4.6% (w/v) TCA, and 0.6% (w/v) iron chloride (FeCl_3_); solution B, 4% 2, 2-dipyridyl (w/v in 70% ethanol). Solutions A and B were mixed in a ratio of 2.75:1 before use. The experiment had three independent biological samples per line.

### Statistical Analysis

The data were analyzed using SPSS version 17.0 and a Student’s *t*-test. Mean and standard error values were calculated for the variables comparison. Values of *P* < 0.05 and *P* < 0.01 were considered statistically significant.

## Results

### *SlBRI1* Is Ubiquitously Expressed

**FIGURE 1:**
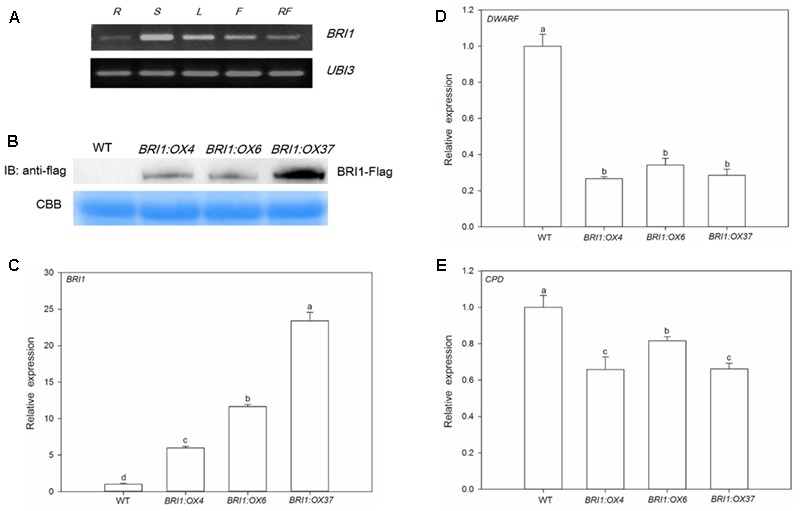
*SLBRI1* expression in wild type (WT) and *SLBRI1-*overexpressing (*BRI1:OX*) plants. **(A)** *SLBRI1* expression patterns in WT. R, Root; S, Stem; L, Leaf; F, Flower; RF, Red Fruit. **(B)** Transgenic BRI1 protein levels examined by immunoblotting with anti-Flag antibodies. CBB, Coomassie brilliant blue. **(C)** The relative transcript levels of *SLBRI1.* **(D)** The relative transcript levels of *DWARF.* **(E)** The relative transcript levels of *CPD.* Data values are the means ± SD of three independent biological samples. Different letters indicate significant differences according to Student’s *t*-test (*P* < 0.05).

*SlBRI1* expression in different organs was determined using RT-PCR, and we observed that expression was very low in roots, higher in flowers and red fruits, and highest in stems and leaves (**Figure 1A**), suggesting that *SlBRI1* is involved in regulating both vegetative and reproductive growth.

### Overexpression of *SlBRI1* Increases the Endogenous BR Signal Intensity

We generated transgenic tomato plants expressing *SLBRI1* under the control of the constitutive CaMV 35S promoter. Transcript levels of *SlBRI1* in the three transgenic lines *BRI1:OX4, BRI1:OX6*, and *BRI1:OX37* were 6.0, 11.6, and 23.4 times higher, respectively, than in WT plants (**Figure 1C**). The expression levels of the BR biosynthetic gene, *DWARF*, in these transgenic lines were 3.7, 2.9, and 3.5 times lower, respectively, than in WT plants for *DWARF* (**Figure 1D**), while those of another BR biosynthetic gene, *CPD*, were 1.52, 1.23, and 1.51 times lower, respectively (**Figure 1E**). Similarly, Western blot analysis indicated that SLBRI1 protein levels in the *BRI1:OX4* and *BRI1:OX6* lines were lower than in the *BRI1:OX37* line (**Figure 1B**), while the SLBRI1-Flag fusion protein was, as expected, not detected in the WT plants. Taken together, these results indicate that overexpression of *SLBRI1* resulted in increased transcription and expression of *SLBRI1* and caused a feedback-inhibition of BR biosynthetic genes, which consequently showed decreased expression. This is consistent with an increase in the intensity of endogenous BR signaling in the *SLBRI1* overexpressing transgenic lines (He et al., 2005).

### Overexpression of *SlBRI1* Causes Precocious Seed Germination and Accelerated Seedling Growth

**FIGURE 2:**
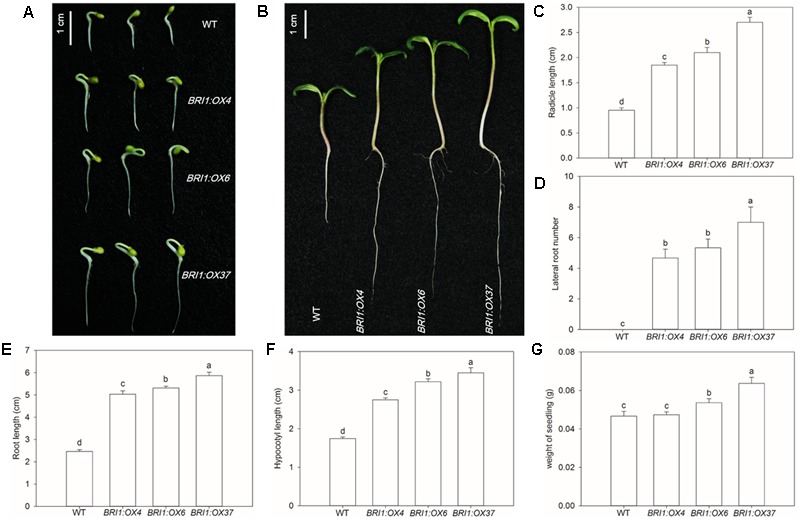
Effects of overexpressing *SLBRI1* on tomato seed germination. **(A)** Phenotypes of germinating seeds at 3 days. **(B)** Phenotypes of germinating seeds at 7 days. **(C)** Radicle length at 3 days. **(D)** Lateral root number at 7 days. **(E)** Root length at 7 days. **(F)** Hypocotyl length at 7 days. **(G)** Weight of seedlings at 7 days. Data values are the means ± SD of 15 independent biological samples. Different letters indicate significant differences according to Student’s *t*-test (*P* < 0.05).

To test whether the *SLBRI1* overexpression mediated increase in BR signaling affected seed germination seeds of WT, *BRI1:OX4, BRI1:OX6*, and *BRI1:OX37* were germinated on agar and were monitored for 7 dyas. *BRI1:OX4, BRI1:OX6*, and *BRI1:OX37* seeds germinated earlier than WT seeds (**Figure 2A**). Additionally, radicle lengths increased in proportion to the increase in *SLBRI1* expression, such that the radicle lengths in the *BRI1:OX4, BRI1:OX6*, and *BRI1:OX37* lines were 1.9-, 2.1-, and 2.7-fold greater, respectively, than in WT, 3 days after germination (**Figure 2C**). Compared with WT, the transgenic lines showed increased root and hypocotyl length, number of lateral roots and seedling weight after 7 days of germination (**Figures 2B–G**), highlighting the critical role of BR signaling in seed germination and early growth vigor.

### Plant Growth and Photosynthesis Are Enhanced in *SLBRI1* Overexpression Lines

**FIGURE 3:**
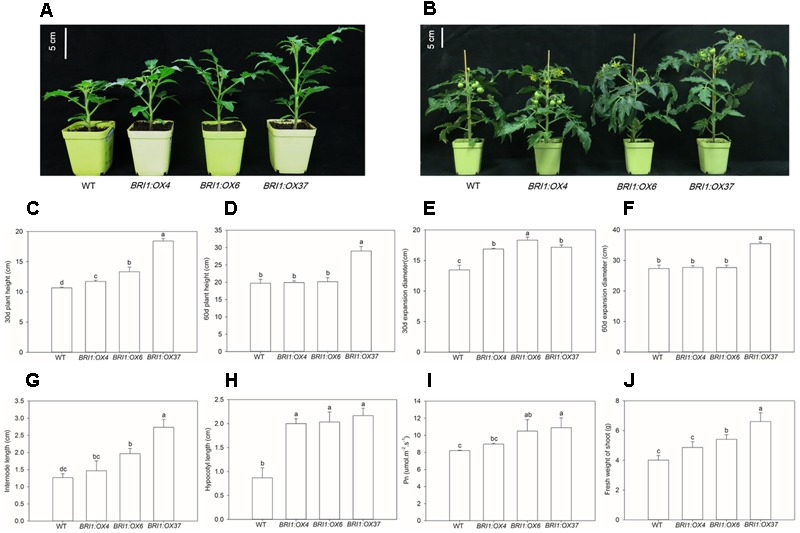
Effects of overexpressing *SLBRI1* on growth and photosynthesis. **(A)** Plant phenotypes at 30 days after sowing. **(B)** Plant phenotypes at 60 days after sowing. **(C)** Plant height at 30 days after sowing. **(D)** Plant height at 60 days after sowing. **(E)** Expansion diameter at 30 days after sowing. **(F)** Expansion diameter at 60 days after sowing. **(G)** Internode length at 30 days after sowing. **(H)** Hypocotyl length at 30 days after sowing. **(I)** Leaf CO_2_ assimilation rate (Pn) at 30 days after sowing. **(J)** Fresh weight at 30 days after sowing data values are the means ± SD of 15 independent biological samples (Pn with five replicates). Different letters indicate significant differences according to Student’s *t*-test (*P* < 0.05).

**FIGURE 4:**
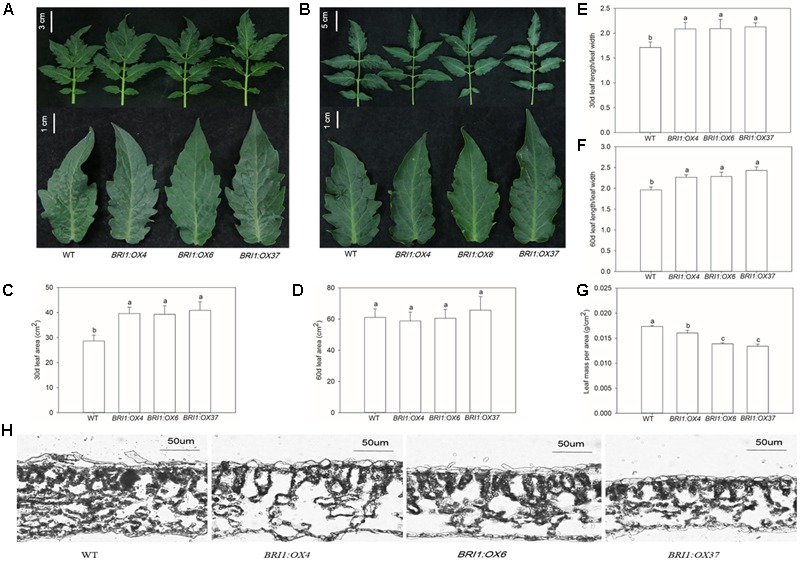
Effects of overexpressing *SLBRI1* on tomato leaf growth. **(A)** The phenotypes of the third leaf at 30 days after sowing. **(B)** The phenotypes of the sixth leaf at 60 days after sowing. **(C)** The area of the third leaf at 30 days after sowing. **(D)** The area of the sixth leaf at 60 days after sowing. **(E)** The length/leaf width of the third leaf at 30 days after sowing. **(F)** The length/leaf width of the sixth leaf at 60 days after sowing. **(G)** The mass per area of the third leaf at 30 days after sowing. **(H)** The structure of the third leaf at 30 days after sowing. Data values are the means ± SD of 15 independent biological samples. Different letters indicate significant differences according to Student’s *t*-test (*P* < 0.05).

**FIGURE 5:**
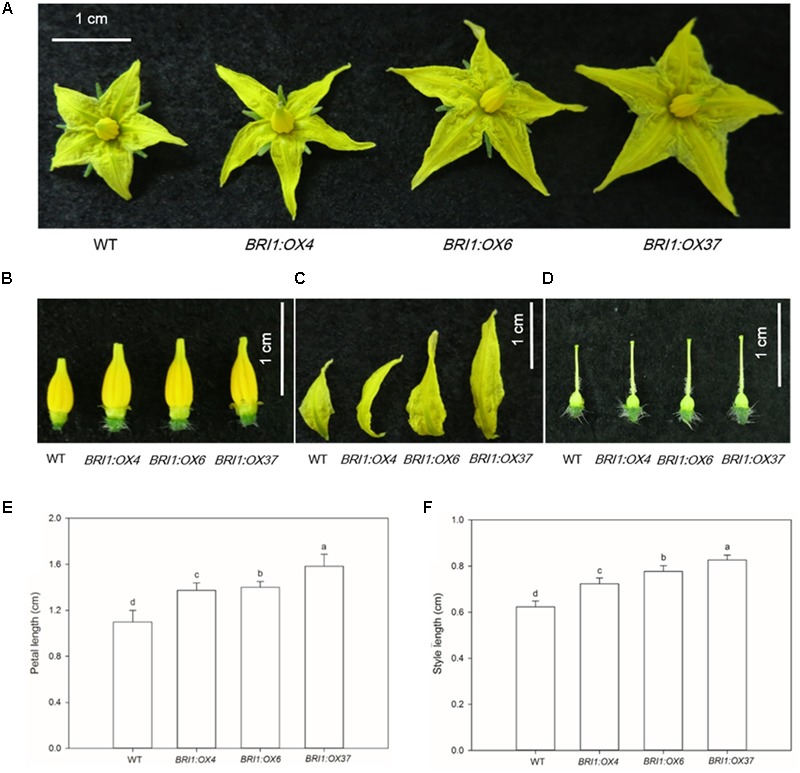
Effects of overexpressing *SLBRI1* on flowers. **(A)** Flower phenotypes at 30 days after sowing. **(B–D)** Stamen, petal, and style phenotypes at 30 days after sowing. **(E)** Petal length at 30 days after sowing. **(F)** Style length at 30 days after sowing. Data values are the means ± SD of 15 independent biological samples. Different letters indicate significant differences according to Student’s *t*-test (*P* < 0.05).

To elucidate the effects of the *SLBRI1* overexpression mediated increase in BR signaling on the growth of tomato plants, we observed the phenotypes of WT, *BRI1:OX4, BRI1:OX6*, and *BRI1:OX37* plants grown in a growth chamber. The transgenic plants exhibited increased plant height, expansion diameter, internode length, and hypocotyl length 30 days after sowing (**Figures 3A,C,E,G,H**), and also 60 days after sowing in the case of the *BRI1:OX37* line (**Figures 3B,D,F**). The transgenic plants had a larger leaf area than WT at the early growth stage (**Figures 4A,C**), and also longer leaves throughout development. The transgenic plants also exhibited a reduced leaf thickness and leaf mass per area compared with WT (**Figure 4**), as well as larger flowers with longer petals and style (**Figure 5**). Finally, gas exchange analyses indicated that the *SLBRI1*-overexpressing plants had an increased CO_2_ assimilation rate and FW 30 days after sowing (**Figures 3I,J**). Taken together, these results indicated that the increase in BR signaling due to *SLBRI1* overexpression promoted various aspects of growth and development, as well as photosynthetic capacity.

### Overexpression of *SlBRI1* Causes an Increase in Ethylene Production and in the Expression of Ethylene Biosynthetic Genes

**FIGURE 6:**
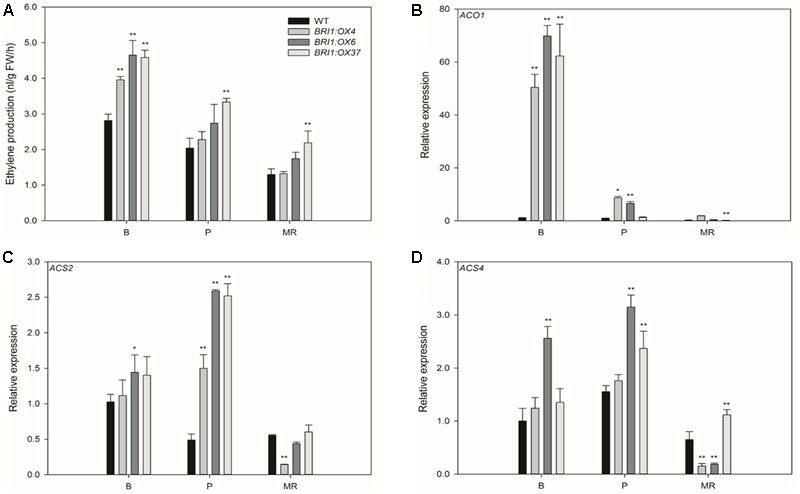
Effects of overexpressing *SLBRI1* on the ethylene production rate and expression of ethylene biosynthesis genes in tomato fruit. **(A)** Ethylene production rate. **(B)** The relative expression level of *ACO1*. **(C)** The relative expression level of *ACS2*. **(D)** The relative expression level of *ACS4*. Data values represent the means ± SD of three independent biological samples. The samples were collected at the same fruit developmental stage. Asterisks indicate significant differences between the transgenic lines and WT (^∗^ *P* < 0.05; ^∗∗^ *P* < 0.01; Student’s *t*-test). B, breaker; P, pink; MR, mature red.

To test whether *SLBRI1* expression level is important for the regulation of ethylene production, we measured ethylene accumulation in the fruits of the transgenic lines and WT by gas chromatography. Ethylene production in *BRI1:OX4, BRI1:OX6*, and *BRI1:OX37* were 65.1, 40.5, and 62.7% higher, respectively, than in WT at the B stage (**Figure 6A**). Ethylene production in the transgenic lines was still higher than in WT up to MR stage. We also measured the transcript abundance of the ethylene biosynthetic genes *ACO1, ACS2*, and *ACS4*, which are important for ethylene production in tomato fruit (Barry et al., 1996; Barry et al., 2000; Klee and Giovannoni, 2011). Compared with WT fruit, *ACO1* and *ACS2* transcript levels in the fruits of the three transgenic lines were markedly higher than in WT at the B and P stages (**Figures 6B,C**), with *ACO1* expression being 69-, 50-, and 62-fold higher at the B stage. Similarly, *ACS4* transcript levels were higher in the transgenic lines than in WT at the B and P stages (**Figure 6D**). These results showed that *SLBRI1* overexpression mediated increases in BR signaling promote ethylene production during tomato fruit ripening by upregulating the expression of ethylene biosynthetic genes.

### Overexpression of *SlBRI1* Leads to Higher Fruit Carotenoid Accumulation and Expression of Lycopene Biosynthetic Genes

**FIGURE 7:**
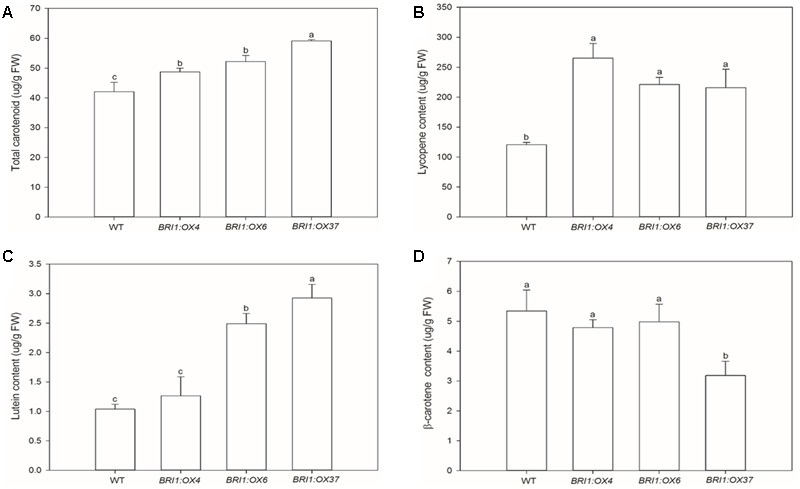
Effects of overexpressing *SLBRI1* on tomato fruit carotenoid contents. **(A)** Total fruit carotenoid contents. **(B)** Fruit lycopene contents. **(C)** Fruit lutein contents. **(D)** Fruit β-carotene contents. The samples were collected at the same MR stage. Data values are the means ± SD of three independent biological samples. Different letters indicate significant differences according to Student’s *t*-test (*P* < 0.05).

To test whether the *SLBRI1* overexpression mediated increase in BR signaling affects carotenoid accumulation, total carotenoid levels were determined by spectrophotometry. We observed that total carotenoid accumulation was significantly greater in the *SLBRI1-*overexpressing lines (**Figure 7A**). Furthermore, when lycopene, β-carotene, and lutein accumulation in the pericarp of fruit at the MR stage from the transgenic lines and WT was measured, using HPLC, the *SLBRI1* transgenic lines showed increased levels of both lycopene and lutein. The lycopene abundance was 120, 84, and 79% greater in the pericarp of *BRI1:OX4, BRI1:OX6*, and *BRI1:OX37*, respectively, than in WT (**Figure 7B**), while the lutein levels were 22, 140, and 182% greater, respectively (**Figure 7C**). We observed no significant difference in β-carotene levels between the transgenic lines and WT (**Figure 7D**). Taken together, these results indicated that carotenoid accumulation in tomato fruits correlated with the BR signaling strength in the *SLBRI1* overexpression lines.

**FIGURE 8:**
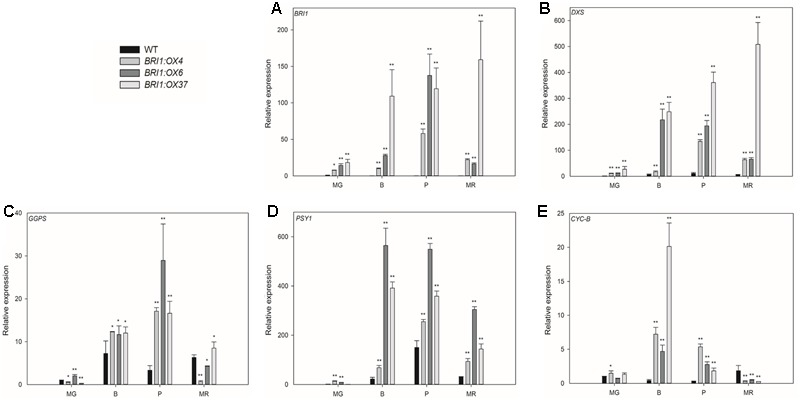
Effects of overexpressing *SLBRI1* on genes involved in the synthesis of carotenoids. **(A)** *SLBRI1* expression. **(B)** *DXS* expression. **(C)** *GGPS* expression. **(D)** *PSY1* expression. **(E)** *CYC-B* expression. Data values represent the means ± SD of three independent biological samples. The samples were collected at the same developmental stage. Asterisks indicate significant differences between transgenic lines and WT (^∗^*P* < 0.05; ^∗∗^*P* < 0.01; Student’s *t*-test). MG, mature green; B, breaker; P, pink; MR, mature red.

To investigate whether changes in the expression of genes in the carotenoid biosynthetic pathway correlated with changes in carotenoid content, we used qRT-PCR to determine the transcript levels of *SlBRI1* (**Figure 8A**), *SlDXS* (**Figure 8B**), *SlGGPS* (**Figure 8C**), *SlPSY1* (**Figure 8D**), and *SlCYC-B* (**Figure 8E**) in the pericarp of fruit at four stages. *SlBRI1* expression in the three transgenic lines was significantly greater at all four stages, with *SlBRI1* transcript abundance in *BRI1:OX37* being 18-, 109-, 119-, and 159-fold higher than in WT at the MG, B, P, and MR stages, respectively (**Figure 8A**). Lycopene biosynthesis related genes (*SlDXS, SlGGPS*, and *SlPSY1*) were also all markedly up-regulated in the three transgenic lines at the B, P, and MR stages, with the exception of *SlGGPS* in the *BRI1:OX4* and *BRI1:OX6* lines at the R stage (**Figures 8B–D**). Additionally, *SlDXS* expression was also significantly up-regulated at the MG stage (**Figure 8B**). *SlCYC-B* encodes an enzyme that catalyzes the conversion of lycopene to β-carotene (Hirschberg, 2001), and its expression in the transgenic lines was higher in the B and P stages compared to WT, but was significantly lower at the MR stage (**Figure 8E**). These results are consistent with previous reports (Römer and Fraser, 2005; Dellapenna and Pogson, 2006), that the expression of genes encoding enzymes involved in carotenoid biosynthesis determining the total levels of carotenoids, and that *SlBRI1* overexpression cause an increase in BR signaling, which is important for carotenoid accumulation.

### Overexpression of *SlBRI1* Accelerates Fruit Ripening and Improves Fruit Quality

**FIGURE 9:**
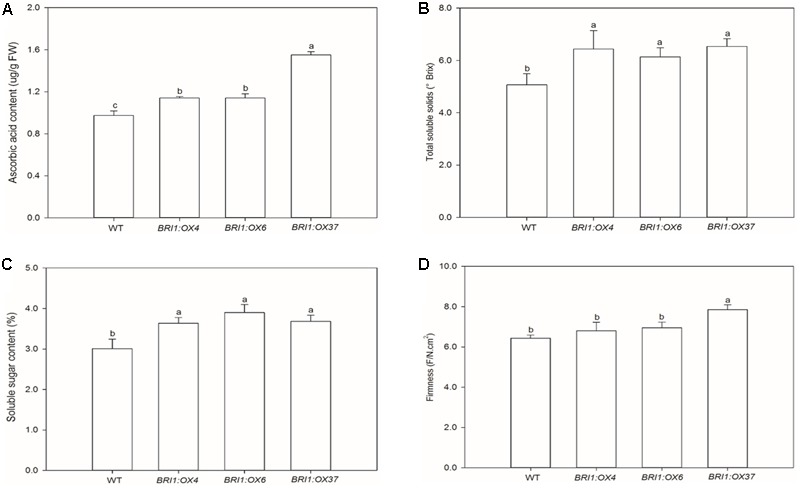
Effects of overexpressing *SLBRI1* on tomato fruit quality attributes. **(A)** Ascorbic acid contents. **(B)** Total soluble solids. **(C)** Soluble sugar contents. **(D)** Fruit firmness. Data values represent the means ± SD of six independent biological samples (Soluble sugar contents with three replicates). The samples were collected at the same MR stage. Different letters indicate significant differences according to Student’s *t*-test (*P* < 0.05).

We also investigated whether the *SlBRI1* expression level affected fruit quality and yield. The transgenic lines exhibited a slight increase in fruit yield and number per plant compared with WT, but a decrease in individual fruit weight. *SlBRI1* overexpression resulted in early flowering and a significant reduction in the ripening time (Supplementary Table S1). Levels of ascorbic acid in the fruit of the *BRI1:OX4, BRI1:OX6*, and *BRI1:OX37* lines were 17, 17, and 59% higher, respectively, than in WT (**Figure 9A**), and the increase in total soluble solids was 27, 22, and 29%, respectively (**Figure 9B**). Furthermore, the contents of soluble sugars in fruit from the *BRI1:OX4, BRI1:OX6*, and *BRI1:OX37* lines were 21, 30, and 22% higher than in WT (**Figure 9C**). The firmness of fruit from the *BRI1:OX37* line was greater than WT, while no difference was observed between the *BRI1:OX4* and *BRI1:OX6* lines compared to WT (**Figure 9D**).

## Discussion

The role of BRs and BR signaling in plant growth and development is well established, based mostly on studies of *A. thaliana* (Zhu et al., 2013). Their role as regulators of growth, development, fruit yield, and quality have also been investigated in tomato (Liu et al., 2014; Li et al., 2015; Zhu et al., 2015). However, little is known about the potential role of *SlBRI1* and BR signaling in regulating tomato architecture or fruit nutrient content. We generated tomato *SlBRI1*-overexpressing plants, which we concluded showed increased BR signaling intensity based on the following evidence: SlBRI1 mRNA and protein levels in the overexpressing plants were significantly higher than in WT (**Figures 1B,C**); the expression levels of the *DWARF* and *CPD* genes were markedly inhibited in the transgenic plants (**Figures 1D,E**) (He et al., 2005); and the hypocotyls and roots of the transgenic plants were significantly longer than WT (**Figures 2E,F**), which is known to closely correlate with the BR signaling intensity (Koka et al., 2000). Taken together, these results indicated that the transgenic plants had increased BR signaling, which resulted in expression of feedback-inhibited BR biosynthetic genes and an increase in hypocotyl and root length.

Known BR mutants include mutants in BR biosynthetic genes causing BR deficiency and in BR receptor genes, causing BR insensitivity, and these mutant types both have a dwarfed phenotype, dark green leaves, and malformed stems and flowers in *A. thaliana* and tomato (Chory et al., 1991; Szekeres et al., 1996; Li and Chory, 1997; Montoya et al., 2002). In contrast, *SlBRI1* overexpression in tomato, as shown here, caused increased plant height, internode length, and hypocotyl length (**Figure 3**). *DWARF* overexpression has been shown to cause an increase in endogenous BR levels and also resulted in increased plant height, but resulted in a more slender and compact plant architecture at later developmental stages (Li et al., 2015). In contrast, we found that *SlBRI1* overexpression enhanced the expansion diameter of the *BRI1:OX37* lines throughout development (**Figure 3**). Earlier studies showed that a mutation in a receptor *AtBRI1* gene in *A.* thaliana resulted in a reduction in petiole and leaf length, whereas overexpression of *AtBRI1* resulted in longer petioles, slender leaves, and increased sensitivity to exogenous BRs (Fujimura and Samet, 2001). We also observed longer petioles and a slender leaf phenotype, as well as decreased leaf thickness (**Figure 4**), which has been seen in *DWARF* overexpressing tomato plants (Li et al., 2015). Furthermore, our *SlBRI1-*overexpressing plants exhibited larger flowers, with longer petals and stigmas than the WT (**Figure 5**). Therefore, the regulatory role of the endogenous BR level and BR signaling in plant growth and development may differ in different organs and developmental stages of tomato. We also found that the net photosynthetic rate and FW increased with an increase in *SlBRI1* expression (**Figure 3**), indicating that the increase in BR signaling caused by *SlBRI1* overexpression is associated with a regulation of photosynthetic capacity in tomato.

Ethylene plays a critical role in the regulation of tomato fruit ripening, and previous studies have shown that BR treatment induces ethylene production by regulating the expression of genes involved in ethylene biosynthesis (Zhu et al., 2015). In addition, overexpression of the *DWARF* gene was also shown to promote ethylene production and ripening in tomato fruit (Li et al., 2015). In this current study, our results indicated that the *SlBRI1* overexpression mediated increase in BR signaling promoted ethylene production and fruit ripening (**Figure 6** and Supplementary Table S1). However, the molecular mechanism underlying the increased endogenous BR levels or BR signaling to promote ethylene production has not yet been investigated. The ethylene biosynthetic genes *ACO1, ACS2*, and *ACS4* play critical roles in regulating ethylene production during fruit ripening (Barry et al., 1996, 2000; Klee and Giovannoni, 2011), and we observed that the mRNA levels of *ACO1* and *ACS2* were significantly higher in the transgenic plants than in WT (**Figure 6**). BRs have also been shown to induce the transcription of *ACS* genes and increase ACS protein stability (Zimmermann et al., 2004; Hansen et al., 2009). We conclude that increased BR levels or BR signaling may up-regulate the expression of ethylene biosynthetic genes and in turn enhance the ethylene production rate, thereby promoting fruit ripening and the transcription of ripening-related genes.

Carotenoid accumulation has been shown to be increased by BRs and decreased by application of the drug Brassinazole (Zhu et al., 2015). In addition, overexpression of the *DWARF* gene was reported to cause an increase in BR levels, thereby enhancing carotenoid accumulation in tomato fruit (Li et al., 2015). Finally, overexpression of *AtBZR1-1D* in tomato, is known to promote carotenoid accumulation by regulating the expression of carotenoid biosynthetic genes (Liu et al., 2014). In this study, we found that *SlBRI1* overexpression markedly increased fruit carotenoid accumulation (except β-carotene) (**Figure 7**). Previous studies have shown that the expression levels of genes encoding the biosynthetic enzymes involved in carotenoid biosynthesis directly regulate carotenoid accumulation (Römer and Fraser, 2005; Dellapenna and Pogson, 2006; Sandmann et al., 2006). Here, we used qRT-PCR to measure the transcript levels of such genes, and found that *SlDXS, SLGGPS*, and *SlPSY1* expression were significantly enhanced in *SlBRI1*-overexpressing plants compared to WT (**Figure 8**). Our results suggested that *SlBRI1* overexpression mediated increases in BR signaling may directly or indirectly up-regulate the expression of carotenoid biosynthetic genes, thereby elevating carotenoid accumulation.

Yield and nutrient quality are important factors in tomato production. We showed that the *SlBRI1*-overexpressing plants had slightly increased fruit yield and fruit number, but lower individual fruit weight (Supplementary Table S1). In contrast, *DWARF* overexpression generated larger fruits, and decreased fruit numbers and fruit yield per plant (Li et al., 2015). These results indicate that enhanced endogenous BR levels or increased endogenous BR signaling had different regulatory effects on fruit size and yield. *SlBRI1* overexpression resulted in early flowering and accelerated ethylene production and ripening in the fruits (Supplementary Table S1), and *DWARF* overexpressing plants showed similar phenotypes (Li et al., 2015). These results indicate that increased endogenous BR levels or BR signaling might both accelerate fruit ripening by promoting ethylene production. Finally, previous studies have shown that application of BRs or *AtBZR1-1D* overexpression in tomato caused improved fruit quality attributes (Liu et al., 2014; Zhu et al., 2015). We observed that *SlBRI1* overexpression resulted in an increase in soluble solids, soluble sugars, and ascorbic acid levels, as well as the fruit firmness, during tomato fruit ripening (**Figure 9**), indicating that *SlBRI1* overexpression mediated increases in BR signaling can promote fruit ripening and quality.

## Conclusion

*SlBRI1* overexpression increased the endogenous BR signaling intensity and improved fruit carotenoid accumulation and quality attributes in transgenic tomato. The increased BR signaling either directly up-regulated the expression of key ethylene biosynthetic genes to promote fruit ripening, or directly up-regulated the expression of carotenoid biosynthetic genes and ripening-related genes. Taken together, our results indicate that endogenous BR signaling plays an important role in regulating growth and fruit nutrient quality in tomato. Therefore, these results underline the potential for improving growth and fruit nutritional quality by genetically manipulating *SlBRI1* expression levels which was feasible in tomato. In addition, the constitutive activation of BR signaling enhanced freezing resistance by regulating cold responsive gene expression in Arabidopsis (Eremina et al., 2016). Further study will confirm increased BR signaling intensity whether can also improve freezing resistance and other stress response in tomato.

## Author Contributions

SN wrote the main manuscript text. SN and XW contributed to the conception of the study. SN, SH, and SW performed the experiments. SN, SH, DC, JL, SL, and QL performed the data analyses and helped perform the analysis with constructive discussions. All the authors gave the final approval for submission of the manuscript.

## Conflict of Interest Statement

The authors declare that the research was conducted in the absence of any commercial or financial relationships that could be construed as a potential conflict of interest.
