# Supplementary material for: Enhancing Brassinosteroid Signaling via Overexpression of Tomato (Solanum lycopersicum) SlBRI1 Improves Major Agronomic Traits
Source: Front Plant Sci. 2017 Aug 10;8:1386. doi: 10.3389/fpls.2017.01386 (PMC5554372; doi:10.3389/fpls.2017.01386)
Supplement: Supplementary file 1 [file Table_1.docx]

**Table S1.** **Overexpression of *BRI1* accelerates fruit ripening and improves yield**

| Parameter | WT | *BRI1:OX4* | *BRI1:OX6* | *BRI1:OX37* |
| --- | --- | --- | --- | --- |
| Fruit number per plant | 19.4±2.4b | 19.6±1.14b | 21.5±1.29b | 24.6±1.14a |
| Fruit weight per fruit (g) | 2.97±0.35a | 2.98±0.2a | 2.78±0.15a | 2.62±0.14a |
| Fruit yield per plant (g) | 57.75±2.86b | 58.32±4.14b | 59.79±1.68ab | 64.4±3.24a |
| Days from anthesis to fruit ripening | 54.25±1.26a | 50.75±1.25b | 48.87±0.85b | 45.5±1.29c |

Data are expressed as average ± SD for 15 plants grown in a growth chamber. Different letters indicate significant differences according to Student’s t-test (P < 0.05).
