# Supplementary material for: Enhancing Brassinosteroid Signaling via Overexpression of Tomato (Solanum lycopersicum) SlBRI1 Improves Major Agronomic Traits
Source: Front Plant Sci. 2017 Aug 10;8:1386. doi: 10.3389/fpls.2017.01386 (PMC5554372; doi:10.3389/fpls.2017.01386)
Supplement: Supplementary file 2 [file Table_2.DOCX]

**Table S2.** **Primers used in this study**

| Gene | Forward primer (5’-3’) | Reverse primer (5’-3’) | Reference |
| --- | --- | --- | --- |
| *ACO1* for Q-PCR  *ACS2* for Q-PCR | TAATGGGAATGGGAAGAAAAGATT | ACAAAGCAAGATAAAGCACCCC | (Wang *et al*., 2014) |
|  | AAGCTTAACGTCTCGCCTGG | CCACCCTGGCTCTTGACATT | (Wang *et al*., 2014) |
| *ACS4* for Q-PCR  *DXS* for Q-PCR | TCAACGTCTCCCCTGGATGGATCT | TGCAAGTGCGATCTCCATTG | (Wang *et al.,* 2014) |
|  | AGCTTCCGGCTGGAAACAAA | CTAGCACAATAGCAGCATCC | (Galpaz *et al*., 2008) |
| *GGPS* for Q-PCR | GTACCTCGCTACCGCTACA | TAATCCCACATTAGGGTTACC | (Galpaz *et al*., 2008) |
| *PSY1* for Q-PCR | AACTTGTTGATGGCCCAAAC | CTGTATCGGACAAAGCACCA | (Galpaz *et al.,* 2008) |
| *CYCB* for Q-PCR | TGTTATTGAGGAAGAGAAATGTGTGAT | TCCCACCAATAGCCATAACATTTT | (Lee *et al.,* 2012) |
| *CPD* for Q-PCR | CTTCTCTCCGAGCTGTTCATCTAG | GAAGGAAAACAGAGAGTTCCACTC | (Peng *et al.,* 2014) |
| *DWARF* for Q-PCR | AAAATTGATGAGTTTATGAGATCCC | CAAGCATATCATGTTGAATTTCCT |  |
| *BRI1* for Q-PCR | TTCAATGGCACGATCCCGAA | TGGGGAGAGGATACCCACAG |  |
| *BRI1* for over-  expression vector | CACGGGGGACTCTAGAATGAAA  GCTCACAAAACTGTGTTTAAC | CGTCCTTGTAGTCCATGGTACC  AAGGTGTTTGCTCAGCTCATTG |  |
| *BRI1* for RT-PCR | TTTGAGTGTCGCCACTCTTGCC | CGTCCTTGTAGTCCATGGTACC |  |
| *UBI3* for Q-PCR | GTGTGGGCTCACCTACGTTT | ACAATCCCAAGGGTTGTCAC |  |
